# Nanomechanical Behavior of Multi-Walled Carbon Nanotubes Particulate Reinforced Aluminum Nanocomposites Prepared by Ball Milling

**DOI:** 10.3390/ma9030140

**Published:** 2016-02-26

**Authors:** Farhad Ostovan, Khamirul Amin Matori, Meysam Toozandehjani, Arshin Oskoueian, Hamdan Mohamed Yusoff, Robiah Yunus, Azmah Hanim Mohamed Ariff

**Affiliations:** 1 Materials Synthesis and Characterization Laboratory, Institute of Advanced Technology, Universiti Putra Malaysia, 43400 Serdang, Malaysia; F.ostovan@gmail.com (F.O.); Toozandejani.meysam@yahoo.com (M.T.); arshin.oskoueian@gmail.com (A.O.); 2 Department of Physics, Faculty of Science, Universiti Putra Malaysia, 43400 Serdang, Malaysia; 3 Department of Chemical and Environmental Engineering, Faculty of Engineering, Universiti Putra Malaysia, 43400 Serdang, Malaysia; my_hamdan@upm.edu.my (H.M.Y.); robiah@upm.edu.my (R.Y.); 4 Department of Mechanical and Manufacturing Engineering, Faculty of Engineering, University Putra Malaysia, 43400 Serdang, Malaysia; azmah@upm.edu.my

**Keywords:** nanocomposites, carbon nanotubes (CNTs), ball milling, nanoindentation

## Abstract

The nanomechanical properties of carbon nanotubes particulate-reinforced aluminum matrix nanocomposites (Al-CNTs) have been characterized using nanoindentation. Bulk nanocomposite specimens containing 2 wt % multiwalled CNTs (MWCNTs) were synthesized by a combination of ball milling and powder metallurgy route. It has been tried to understand the correlation between microstructural evolution particularly carbon nanotubes (CNTs) dispersion during milling and mechanical properties of Al-2 wt % nanocomposites. Maximum enhancement of +23% and +44% has been found in Young’s modulus and hardness respectively, owing to well homogenous dispersion of CNTs within the aluminum matrix at longer milling time.

## 1. Introduction

Aluminum matrix composites (AMCs) have found considerable applications in aerospace, automotive and transportation industries owing to its excellent combination of properties such as high strength and stiffness, good wear resistance and improved thermal and electrical properties as compared to the unreinforced aluminum alloys. AMCs are attractive in engineering applications for weight critical applications which may assist in energy saving along with reduction in the cost [1,2,3,4].

Like other metal matrix composites (MMCs), the improvement of mechanical properties of AMCs is closely related to the amount and type of reinforcement as well as the synthesizing process of the composites. With a suitable reinforcement selection for the AMCs, dispersion strengthening can be an effective method for improving their mechanical response. It is well known that addition of carbon nanotubes (CNTs) improves the mechanical properties of CNT reinforced aluminum matrix nanocomposites (Al-CNTs). Different enhancements in the mechanical properties of these nanocomposites have been reported which seems to be scattered. In fact, the intrinsic mechanical properties of the CNTs, CNT content as well as synthesizing procedure of these nanocomposites contribute to the scatter in enhancements [5,6,7,8,9].

Reinforcing of aluminum matrix particularly with those in nano-sized range, necessitate a proper technique which yield a uniforms dispersion of reinforcements. Ball milling process as a part of powder metallurgy route has been employed to disperse reinforcements homogeneously in the metallic matrix particularly aluminum matrix [10,11]. Ball milling as a powder processing method has been reported to be an effective method to provide homogeneous dispersion of the reinforcements within the aluminum matrix [10,11,12,13]. In the case of Al-CNTs, ball milling has been used to effectively incorporate CNTs within aluminum matrix. What is apparent is that within ball milling of Al-CNTs nanocomposite powders, microstructure significantly changes which consequently affects the final mechanical properties of Al-CNT nanocomposites. A uniform dispersion of CNTs and avoiding the agglomeration and clustering of CNTs within the aluminum matrix is the key in obtaining the optimum properties [5,6,9,14,15].

Therefore, in the current work, Al-CNT nanocomposite powders have been synthesized using ball milling and further consolidated in a powder metallurgy route. The main objective is to investigate the contribution of the microstructural evolution within ball milling particularly CNTs dispersion on the micro and nanomechanical properties including Young’s modulus and hardness.

## 2. Results and Discussion

**Figure 1:**
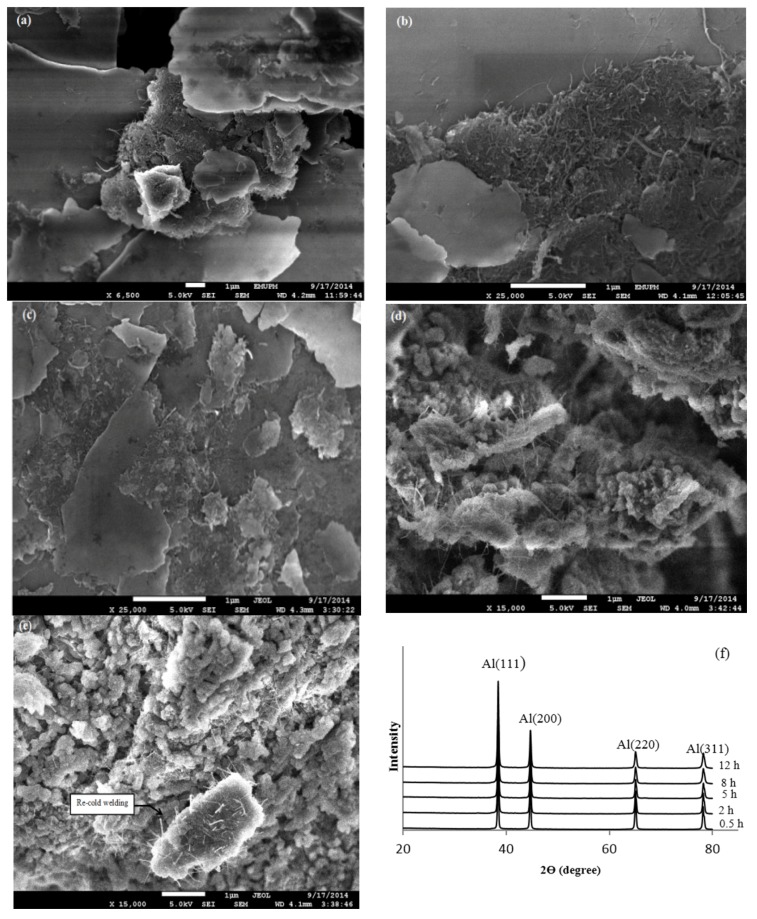
Dispersion uniformity of carbon nanotubes (CNTs) within aluminum matrix in Al-2 wt %CNTs composites milled for (**a**) 0.5 h; (**b**) 2 h; (**c**) 5 h; (**d**) 8 h; (**e**) 12 h and (**f**) X-Ray Diffraction (XRD) patterns of Al-2 wt %CNTs nanocomposites milled for different times.

Figure 1 depicts microstructural evolution of pure Al-2 wt %CNTs nanocomposites as a function of milling time. Shorter milling time of 0.5 h was found as a simple mixing stage which has no considerable effect on dispersion of CNTs within the pure aluminum matrix. It can be observed that the most of CNTs have preserved their initial shapes and remained clustered while a plenty of the surface areas of aluminum particles are uncovered by CNTs (Figure 1a). As milling time increases to 2 h, most of the CNTs are still remained as entangled clusters. It is deduced that at current stage, the form of entangled clusters is partially changed to open entangled. In fact, dispersion of CNTs at shorter milling time is difficult due to the small size of CNTs used (20 nm) which consequently contributes to the poor mechanical properties.

By further increasing the milling time to 5 h more CNTs are dispersed within aluminum particles. Besides, the shortness of CNTs is observed as shown in Figure 1c. A considerable change in the dispersion uniformity of CNTs was observed after 8 h of milling as compared to 5 h of milling. As milling time proceeds, CNTs are separated and shortened to a greater extent and effectively dispersed over surface of the deformed aluminum powders by the progress of cold welding of the deformed powders and fracture of cold welded particles and CNTs finally start to be embedded within the pure aluminum matrix [6,16,17,18]. The well homogenous dispersion of CNTs can be clearly identified after 8 h of milling indicating the efficiency of milling time (Figure 1d). Milling time more than 5 h is long enough to homogenously disperse 2 wt % of CNT within the pure aluminum matrix. Increasing the milling time to 12 h has no significant change in the dispersion of CNTs within the aluminum powder (Figure 1e). However, irregular shape of particles can be observed and this might be due to the high impact of milling after 12 h which causes fracture of the powders after a long time shearing action. Re-cold welding of particles was also observed after 12 h of milling. Cold welded particles are zones where the cracks start to be propagated in the sintered composites [9,18] resulting in drop of the mechanical properties.

On the other hand, no formation of unwanted phases like Al_4_C_3_ phases or aluminum oxides was observed after both ball milling and sintering processes as confirmed by absence of any peak in the X-ray diffraction (XRD) patterns of Al-2 wt %CNTs nanocomposites (Figure 1f). It can be argued that current milling conditions introduce less severe structural damages to CNTs. Therefore, in the current work the effect of these carbide phases (Al_4_C_3_) on the mechanical properties of nanocomposites is not discussed [9,19]. From the mechanical point of view, presence of Al_4_C_3_ is not such detrimental due to the fact that these carbide phases enhance Al–CNT bonding and also hinder CNT pull-out as reported by Esawi *et al.* [5,18].

**Figure 2:**
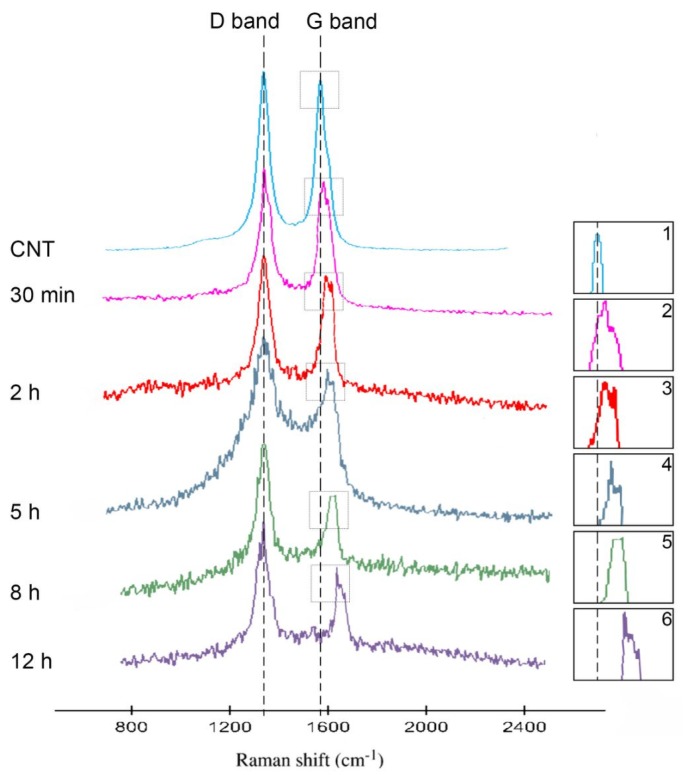
Raman spectra of Al-2 wt % CNT powders as a function of milling time.

A complementary data on the evolution of multiwalled CNTs (MWCNTs) structure with respect to different milling time is provided by Raman spectroscopy (Figure 2 and Table 1). Figure 2 illustrates the Raman spectra of both as-received and milled MWCNTs. Two peaks were detected; the first one at ~1350 cm^−1^ which is representative of D-band and the other at ~1567 cm^−1^ which is representative of G-band. In fact, the I_G_/I_D_ ratio has been defined as a defect density in the graphitic structures [20].

According to Table 1, the as-received MWCNTs show the highest I_G_/I_D_ ratio of 0.97. As milling time proceeds, the I_G_/I_D_ ratio progressively decreases from 0.95 at 30 min of milling to the minimum of 0.55 at 12 h of milling of powders. Therefore, the quantity of defects increases in the MWCNTs structure especially for those powders milled for 12 h. Furthermore, it can be observed that G-band of the MWCNTs milled for 30 min and 12 h are shifted to a large wavenumber, attributing to the residual strain in the MWCNTs [19]. The strains are believed to originate from mechanical alloying which affect CNTs structure. According to Thomson *et al.* [20], this residual strain causes the interatomic distances of the MWCNTs and vibrational frequencies of some of the normal modes to be varied causing Raman peak to be shifted. It is also reported larger strain in the MWCNTs induces larger shift in Raman peak [20]. The higher residual strain in the ball-milled MWCNTs is evident from the G-band shift of the 30 min and 12 h of ball-milled MWCNTs. The lower G-band shift in the 30 min ball-milled MWCNTs might be due to the lower mixing forces at 30 min because of less numbers of interactions between balls and powders at this time. Moreover, the G-band of ball-milled MWCNTs is shifted towards higher wavenumbers, broadened and decreased in amplitude. The intensity of the Raman spectra of 12 h ball-milled MWCNT decreases due to the dilution effect or good dispersion effect of MWCNTs. This indicates that the fraction of embedded CNTs in the soft Al matrix for composites milled for 12 h is relatively higher than of those milled for 30 min. Thus, a better dispersion of MWCNTs could be obtained at 12 h of milling when compared with that of the 30 min of milling time.

**Figure 3:**
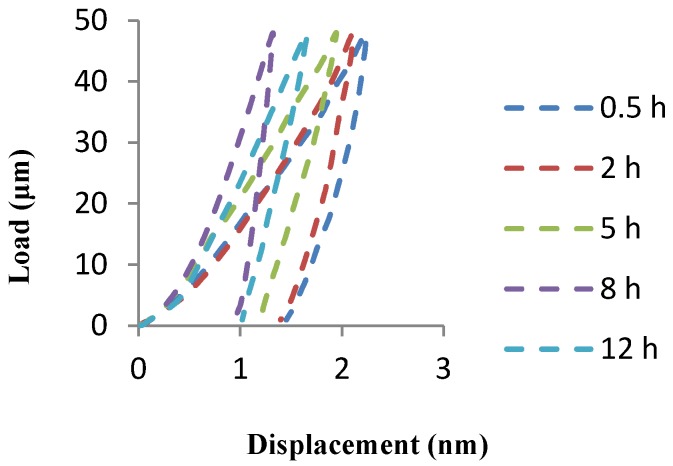
Load–displacement curves of Al-2 wt %CNTs nanocomposite specimens obtained at a peak load of 100 mN at room temperature.

Figure 3 illustrates a representative load *versus* displacement curves for Al-2 wt % CNTs as a function of milling time. Elastic modulus is a function of the slope of the unloading curve in nanoindentation measurements. The plot shows that specimen milled for 0.5 h has the maximum displacement indicating softer matrix and consequently the least Young’s modulus. Whereas, specimen milled for 8 h has the least displacement attributing to the highest Young’s modulus under the same indentation conditions. It is believed that the lower displacement or in other word lower indention depth indicating the strengthening of matrix in the presence of CNTs [21]. Here, the curve shifts toward left and lower displacements as a result of strengthening of matrix due to severe milling condition at longer milling time.

**Figure 4:**
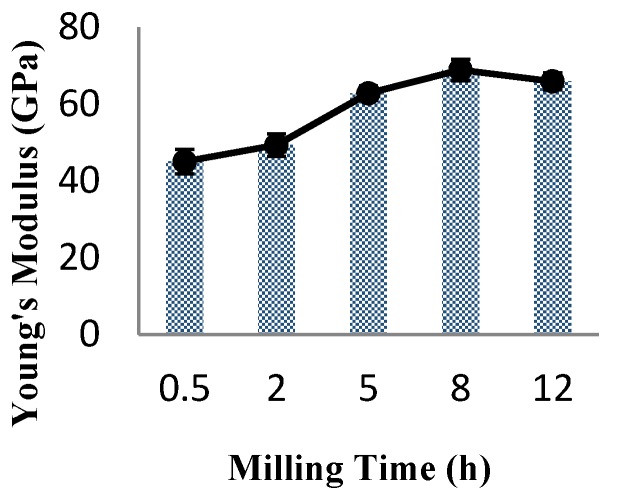
The variation of Young’s modulus of nanocomposite specimens as a function of milling time.

The variation of Young’s modulus of nanocomposite with milling time is shown in Figure 4 and Table 2. It is clear that, as milling time increases up to 8 h, the corresponding Young’s modulus of nanocomposites increases to maximum value of 68.9 GPa. While, further milling up to 12 h decreases the Young’s modulus marginally to 65.9 GPa (Table 2).

Presence of CNT clusters at early stage of milling which act as the precipitates results in the lower Young’s modulus of the nanocomposites. The increase in Young’s modulus values with increasing the milling time attributes to the homogeneous dispersion of CNTs within the pure aluminum matrix which consequently strengthens the matrix along with the grain refining of aluminum powders. Here, specimens milled for 8 h show 22% increase in Young’s modulus which indicating the effective milling procedure in uniform dispersion of CNTs within the matrix. Uniformly dispersed CNTs within the aluminum matrix behave as a homogeneous structure resulting in the enhancement of the Young’s modulus of the Al-2 CNTs nanocomposites [22]. The value of Young’s modulus decreases by increasing the milling time to 12 h. The decrease in the Young’s modulus after 8 h of milling is probably due to the re-cold welding of particles as reported by Esawi *et al.* [5].

**Figure 5:**
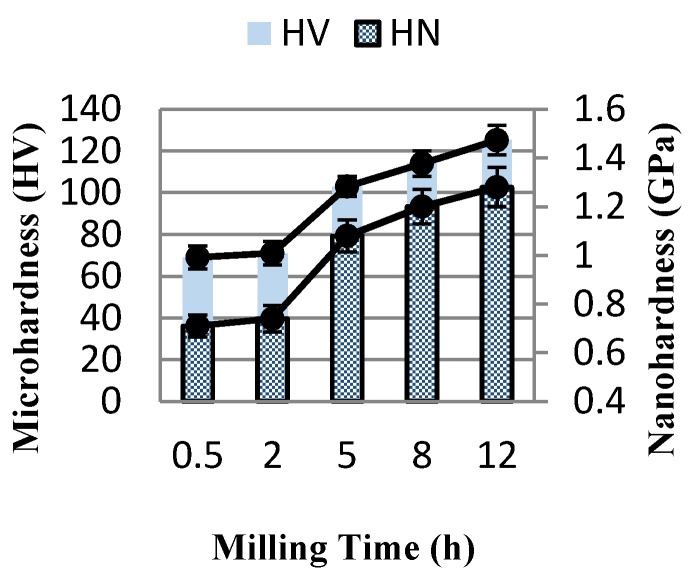
The variation of Nanohardness and microhardness of nanocomposite specimens as a function of milling time.

The nanohardness (HN) values of the Al-2 wt %CNTs nanocomposites were determined at the maximum load in the load–displacement curve as shown in Figure 5 and Table 2. HN values vary in the same manner of microhardness (HV) values (Figure 5). Both values of micro and nanohardness increase as milling time increases up to 12 h where the maximum values are achieved. It is worth to note that both HV and HN values start to increase sharply after 2 h of milling when a significant grain refining occurs. HN and HV values of nanocomposites are found to be enhanced by ~44% after 12 h of milling.

Similar to Young’s modulus, the lower values of both HV and HN at shorter milling time is due to the presence of CNT clustered areas which contributes to poor sintering of specimens. These CNT clustered areas also result in poor consolidation and large amount of porosities in the final bulk specimens as a result of poor diffusion bonding among powders within sintering processes. As milling time proceeds, CNTs are repeatedly fractured, refined and uniformly dispersed within aluminum matrix which contributes to the increase in the hardness values of nanocomposites [6,23,24,25,26]. The higher values of hardness are evidenced by the well dispersion of refined CNTs and least amount of CNT clusters at longer milling time on the surface of deformed aluminum powders (Figure 1b). According to Perez-Bustamante *et al.* [27,28], uniform dispersion of CNTs within the matrix effectively inhibits dislocation movement which consequently increases mechanical properties.

Harsh milling condition at longer milling time, however, well disperse CNTs within matrix but introduces severe strain hardening of the matrix powders through generation of a high amount of dislocation density in powder particles resulting from the matrix deformation. In addition, the higher hardness values might attribute to grain refining which can be accelerated in the presence of high dislocation density due to the interaction between CNTs and dislocations [6]. As milling time increases, strain hardening increases contributing to the further strengthening of the nanocomposites. However, strain hardened matrix powders cause difficulties in further processing of powders in a powder metallurgy route [5]. It is worthy to note that there are some mechanisms which may simultaneously be operated leading to enhancement of hardness and Young’s modulus of the Al-2 wt %CNTs nanocomposites. These mechanisms are namely; Orowon strengthening [29], solid solution hardening [23,24,30], work-hardening [31,32], grain and substructure strengthening, quench hardening and thermal mismatch [6,33].

## 3. Materials and Methods

### 3.1. Materials

**Figure 6:**
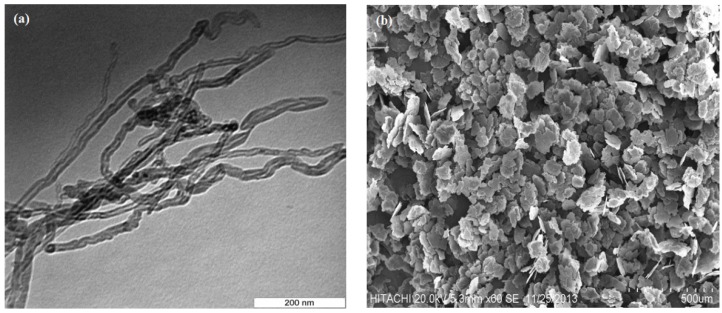
Microstructure of as received materials (**a**) Multiwalled CNTs (MWCNTs) and (**b**) Aluminum powder.

Commercial pure aluminum powder (Merck, Darmstadt, Germany, 99% purity) with an average particle size less than 50 μm and MWCNT powders (Sigma Aldrich, St. Louis, MO, USA, 20–30 nm particle size nm of outer diameter and length of 10 to 30 μm) were used as raw material (Figure 6). The Al-CNT nanocomposites containing 2 wt % multiwalled CNTs (MWCNTs) were synthesized in a combination of ball milling and powder metallurgy route. The MWCNT powders were first dispersed in the pure aluminum matrix in the presence of Stearic acid (2 wt %) using a planetary ball milling machine (PM100, Retsch, Haan, Germany) at room temperature. Stearic acid as a process control agent (PCA) hinders excessive cold welding of the particles during milling process. The ball to powder weight ratio (BPR) of 8:1 was chosen. The powder mixtures were ball milled for different times from 30 min up to 12 h at constant rotational speed of 300 rpm. Nanocomposite powders were then consolidated by cold pressing at a pressure of 150 MPa under argon atmosphere followed by sintering at 530 °C for 45 min under argon atmosphere to obtain sound bulk nanocomposites specimens.

### 3.2. Microstructural Characterization

Before the microstructural characterization, bulk nanocomposite specimens were grinded (with abrasive papers from 400 to 1500 grit), polished (using 6 and 1 μm diamond paste and final polishing using 0.05 μm colloidal silica). Later, dispersion uniformity of CNTs of as received and nanocomposite powders were analyzed through transmission electron microscopy (TEM), scanning electron microscopy (SEM) (S-3400, Hitachi, Dallas, TX, USA), field emission scanning electron microscopy (FESEM) (7600F, JEOL Inc., Peabody, MA, USA). Phase analysis of specimens during milling and after sintering was carried out using X-ray diffraction (XRD). A SHIMADZU XRD-6000 type (Shimadzu Corporation, Osaka, Japan) using 0.1542 nm Cu Kα 181 radiation at 4.8 kW with the voltage of 40 kV and a current of 120 mA were used.

Raman spectra of initial MWCNTs and Al-CNTs mixtures milled for different times up to 12 h were recorded with a Raman spectrometer (Alpha 300R, WITec, Ulm, Germany) with an excitation wavelength of 532.200 nm from a tunable argon laser focused on the sample in order to characterize changes in the MWCNTs structure during milling.

### 3.3. Hardness and Nanoindentation Measurements

Microindentation hardness measurement was carried out on the polished cross-section of nanocomposite specimens using a Mitutoya HV-112 vickers hardness testing machine (Mitutoya, Aurora, IL, USA) at a load of 100 gf and dwell time of 10 s. The average of ten hardness measurement was reported for hardness values. Nanoindentation measurement was conducted using Micro Materials Nanotest™ indenter (Micro Materials, Wrexham, UK) equipped with a standard Berkovich geometry tip indenter in order to deduce the nanohardness (HN) and Young’s modulus (*E*) of nanocomposites. A constant loading rate of 0.5 μN/s was applied to the surface of specimens until reaching the maximum load of 50 μN and holding for 5 s at the maximum load and then unloaded gradually to zero. Total 20 indents were made at different points of specimens for each value of the peak load. Indentation load (*P*) *versus* penetration depth (*h*) curve was generated during both loading and unloading of each specimen. The average values of HN and E modulus were calculated and reported. The values of HN and *E* of nanocomposite specimens were derived according to Oliver–Pharr (O–P) method [34].

## 4. Conclusions

By correlating strength properties and microstructural evolution during ball milling, the following conclusions can be inferred:

1. Dispersion of CNTs within the matrix plays a prominent role on enhancement of mechanical properties of nanocomposite specimens depending on the degree of dispersion within matrix.
2. Uniform dispersion of CNTs at longer milling time along with grain refining enhances the elastic modulus (+23%) and hardness (+44%) of nanocomposite specimens. While, the presence of CNT clusters at shorter aging time contributes to the lower values of hardness and Young’s modulus.

## Figures and Tables

**Table 1:** Raman spectra characteristics of different milled powders as a function of milling time.

Milling Time (h)	As Received	0.5	2	5	8	12
I_G_/I_D_	0.97	0.95	0.82	0.74	0.59	0.55
Position of G-band	1567	1575	1585	1605	1621	1655

**Table 2:** Mechanical properties of Al-CNT composites milled for different times.

Composite	Milling Time (h)	Micro Hardness (HV)	Nano Hardness (GPa)	Young’s Modulus (GPa)
Al-2 wt %CNTs	0.5	69	0.71	45
	2	71	0.74	49.3
	5	103	1.08	62.8
	8	114	1.2	68.9
	12	125.2	1.28	65.9
